# Digital teaching and learning of surgical skills (not only) during the pandemic: a report on a blended learning project

**DOI:** 10.3205/zma001361

**Published:** 2020-12-03

**Authors:** Carina Bachmann, Ana Lucia Paz Hernandez, Stefan Müller, Sadaf Khalatbarizamanpoor, Tino Tschiesche, Frank Reißmann, Lars Kiesow, Daniel Ebbert, Waldemar Smirnow, Arne Wilken, Uta Dahmen

**Affiliations:** 1 Jena University Hospital, Department of General, Visceral and Vascular Surgery, Experimental Transplantation Surgery, Jena, Germany; 2 University of Jena, University Computer Centre, Multimedia Centre, Jena, Germany; 3 ELAN e.V., Osnabrück, Germany

**Keywords:** COVID-19, medical education, practical skills, technology

## Abstract

Due to the COVID-19 pandemic, digital teaching approaches should be used wherever possible. In this article we report on our project for digital teaching and learning of surgical skills.

The compulsory elective “Intensivkurs Chirurgische Techniken” for medical students starting with semester 5 was designed as a blended learning course. One week before the face-to-face class, the students receive the learning and teaching material online in a Moodle course. In the face-to-face class, live demos of procedures (e.g. performing skin and intestinal sutures) are presented by the teachers. The learners then perform the practical procedures and record themselves with the camera of an iPad. They publish their videos in the Moodle course via an Opencast plugin. The implementation of an annotation tool enables everyone in the Moodle course to add free-text comments to selected parts of the videos (video-assisted feedback and coaching).

As a result of the pandemic, the face-to-face class is being moved to a digital learning environment. For this purpose, we are extending the existing system with a web conference tool (BigBlueButton).

## Zusammenfassung

Vor dem Hintergrund der COVID-19-Pandemie sollen Lehrveranstaltungen nach Möglichkeit digital stattfinden. In diesem Beitrag berichten wir über unser Lehrprojekt zum digitalen Lehren und Lernen chirurgischer Fertigkeiten.

Die Wahlpflichtveranstaltung „Intensivkurs Chirurgische Techniken“ für Medizinstudierende ab dem 5. Semester wurde in einem Blended-Learning-Format konzipiert. Eine Woche vor Beginn der Präsenzphase werden den Studierenden die Lernmaterialien für die Veranstaltung in einem Moodle Kurs zur Verfügung gestellt. In der Präsenzphase werden Übungen zu Haut- und Darmnähten in Live-Demos durch die Dozierenden präsentiert. Anschließend führen die Lernenden die praktischen Übungen durch und zeichnen sich dabei mit der Kamera eines iPad auf. Mithilfe eines Opencast-Plugins veröffentlichen sie ihre Videos im Moodle Kurs. Die Einbindung eines Annotation Tool ermöglicht allen Teilnehmenden im Moodle Kurs einzelne Stellen der Videos mit Freitext zu kommentieren (videogestütztes Feedback und Coaching).

In Folge der Pandemie soll nun auch die Präsenzphase digital durchgeführt werden. Dafür wird das vorhandene System um eine Webkonferenzlösung (BigBlueButton) erweitert.

## 1. Introduction

In view of the COVID-19 pandemic, medical educators are facing considerable challenges. Social distancing and other measures to curb the pandemic are severely disrupting traditional practices such as classroom-based teaching [1].

An opportunity is the transition to teaching in a digital environment. Using currently available technologies, this transformation can often be achieved quite easily. Lectures, for instance, are being streamed online or replaced by screencasts (PowerPoint presentation including audio commentary); and seminars delivered as interactive webinars using video conference tools. More challenging for educators, however, are courses on clinical skills training [2]. In order to successfully implement such courses in an online environment, new technical developments play a crucial role.

In the following we report on our project for digital teaching and learning of surgical skills. The aim of the project is to develop a purely virtual, competence-based training of surgical suturing and knotting techniques.

## 2. Description of the project

Our project is a compulsory elective for medical students in the second stage of the programme (“Intensivkurs Chirurgische Techniken”). This 1-week elective is held twice a year in the summer semester break and the winter semester break each for 2 x 25 students.

### 2.1. Didactical concept

The didactical concept builds on “video-based self-reflection and feedback to learn surgical skills” [3], [4]. While performing the practical procedures, the learners video record themselves. On the basis of the videos and feedback they then can thoroughly reflect on their own performance.

#### 2.2. Overall learning objective

The overall learning objective of the course is the acquisition of basic surgical skills on NKLC (national competence-based catalogue of learning objectives in surgery) competence level 3 [5]. This comprises giving the indication for the appropriate suturing technique, carrying out the procedure under supervision, as well as the systematic analysis of one’s own performance including the development of correction strategies. For assessing the learning outcomes a mini-OSCE (1 station) is used.

#### 2.3. Previous structure of the course

The course was designed in a blended learning model. One week before starting the face-to-face class, relevant learning materials (text documents and instructional videos) are supplied to the students in a Moodle course. During the face-to-face class, the teachers first present live demos of the practical procedures, e.g. skin suturing on pig feet or anastomosis techniques on pig intestine. Afterwards, the learners perform the procedures by themselves and video document the process with the camera of an iPad. They upload their videos via an Opencast plugin in Moodle. The videos are hosted on Opencast (open source software for planning, recording, and publishing audiovisual learning content). With the integration of Moodle and Opencast, the videos are available to all participants (teachers and students) in the Moodle course. For analysing the videos an annotation tool is installed in Opencast. This enables course participants to set time markers in the videos and add free-text comments on their own performance or that of others (video-assisted feedback and coaching). The comments are viewable to everyone in the annotation mode in Opencast and can be replied back by the student performing the procedure.

Since face-to-face learning in the classroom is currently being disrupted due to the pandemic, we are modifying the structure of the course.

#### 2.4. Extension of the used system

We are extending the used system to deliver a web-based, virtual suturing and knotting course. For this, the open source web conference tool BigBlueButton (BBB) is being adapted for use and integrated into the system. This BBB implementation enables the live video transmission of both the demos and the students’ performance on the practical procedures in a web conference. Each video track is recorded and then published via Opencast as before. Furthermore, the integration of BBB provides a variety of ways for course (web conference) participants to interact with each other. The students can ask the teachers questions in an easy and direct way via microphone (and camera) or chat [6]. The teachers, on the other hand, can directly observe the students while they are performing the procedural skills and then provide support or intervene in the procedure using the same communication methods.

However, delivering the course in the presented scenario requires a greater logistical effort since the training materials must be available to the students in advance.

## 3. Conclusion

The health and wellbeing of those involved in the learning process is of major importance [7]. To avoid a potential negative impact (for example, on patient safety), clinical skills training, however, should not be abandoned during the pandemic.

Based on our previous developments, we have implemented a blended learning surgical skills course. We are now developing the technical standards for running the whole course in a digital learning environment by extending the system.

It remains to be evaluated on how the students accept the new course and to what extent it can be established as an alternative to traditional classroom instruction after the pandemic resolves.

## Competing interests

The authors declare that they have no competing interests. 
